# A life-threatening complication due to pulmonary haemorrhage following hump-nosed viper bite

**DOI:** 10.1186/s12890-020-1070-9

**Published:** 2020-02-07

**Authors:** Arthihai Srirangan, Jagath Pushpakumara, Kamani Wanigasuriya

**Affiliations:** 1 University Medical Unit, Colombo South Teaching Hospital, Kalubowila, Colombo, Sri Lanka; 2 Department of Medicine, Faculty of Medical Sciences, University of Sri Jayewardenepura, Gangodawila, Nugegoda, Sri Lanka

**Keywords:** Hump-nosed viper, Pulmonary haemorrhage, Methyl-prednisolone

## Abstract

**Background:**

Hump-nosed viper bite, the commonest venomous snake bite in Sri Lanka, is associated with significant morbidity. Specific anti-venom is not available for hump-nosed viper envenomation which is usually managed with supportive treatment. Pulmonary haemorrhage is an unusual manifestation of hump-nosed viper bite. Here we present a case of hump-nosed viper envenomation which complicated by pulmonary haemorrhage and was successfully treated with systemic steroids. To the best of our knowledge, it has not been reported in the literature before.

**Case presentation:**

A previously healthy 55-year-old man presented to the local hospital 18 h after a hump-nosed viper bite. He developed bilateral severe pulmonary haemorrhages, evidenced by rapid desaturation which needed intubation and mechanical ventilation, bleeding from the endotracheal tube and bilateral alveolar shadows in a chest x-ray. He had no other bleeding manifestations. Because of the life-threatening situation, he was treated with methylprednisolone pulse therapy. There was a rapid improvement of hypoxia with a resolution of x-ray changes. He was successfully weaned off from the ventilation after 24 h.

**Conclusion:**

This case highlights the importance of suspecting pulmonary haemorrhage in a patient who develops desaturation and alveolar shadow following hump-nosed viper bite even in the absence of other bleeding manifestation. Early and timely treatment with systemic steroid can be lifesaving in such patients.

## Background

Hump-nosed viper (HNV) bite is one of the medically important envenomation in the Indian subcontinent which causes significant morbidity and mortality [1]. It can cause a variety of clinical manifestations such as local inflammation, blistering and systemic envenomation such as haematological manifestations and acute kidney injury [2].

Although coagulopathy is reported frequently pulmonary haemorrhage is an unusual manifestation of hump-nosed viper bite [3, 4]. Here we report a patient who developed pulmonary haemorrhage which was successfully treated with systemic steroids. This highlights the importance of suspecting pulmonary haemorrhage in a patient who de-saturate with alveolar shadows in chest x-ray despite the absence of overt coagulopathy, and the rapid response to methyl prednisolone.

## Case presentation

A 55-year-old previously healthy man from the western province of Sri Lanka was admitted to Colombo South Teaching Hospital (CSTH) 18 h after an HNV bite. He was initially managed in a peripheral hospital and later transferred as he was anuric for 8 h. He also had vomiting and loose stools. Fang marks were seen on the fifth left toe with pain, minimal swelling and two blisters on the dorsum of the foot. The killed snake was identified by the medical officer as an HNV. He was conscious and rational with a pulse rate of 100 bpm, blood pressure 150/100 mmHg and oxygen saturation of 98%. There was no bleeding tendency or neurological manifestations. Bedside whole blood clotting time was less than 20 min on admission to the peripheral hospital and at 18 h when seen at the CSTH. Polyvalent antivenom was not given as it is ineffective in neutralizing HNV toxicity and carry a high risk of side effects.

Initial investigations revealed, haemoglobin 13.2 g/dl, white cells 13.2 × 10^9^/L, platelets 68 × 10^9^/L, serum sodium 143 mmol/L, serum potassium 4.2 mmol/L and serum creatinine 3.2 mg/dl. On the 2nd day, haematological investigations revealed, haemoglobin 10.5 g/dl, white blood cells 14.1 × 10^9^/L and platelets 58 × 10^9^/L, whole blood clotting time > 20 min, PT/INR 1.7 (reference range: < 1.1) and APTT 48 s (30–40s). Total bilirubin 62.14 μmol/L (5–21) with direct bilirubin 10.08 μmol/L (< 3.4), serum alanine aminotransferase (ALT) 171 U/L (10–40), serum aspartate aminotransferase (AST) 808 U/L (10–35), Creatine kinase (CK) 750 U/L (15–105), serum lactate dehydrogenase (LDH) 2370 U/L (230–460) and serum creatinine was 409 μmol/L (70–120). Blood picture revealed fragmented red cells and thrombocytopenia suggestive of microangiopathic haemolytic anaemia (MAHA). In view of thrombotic microangiopathy (TMA), he was transfused with fresh frozen plasma with the improvement of INR and APTT. The patient was commenced on haemodialysis due to acute kidney injury (AKI).

**Fig. 1:**
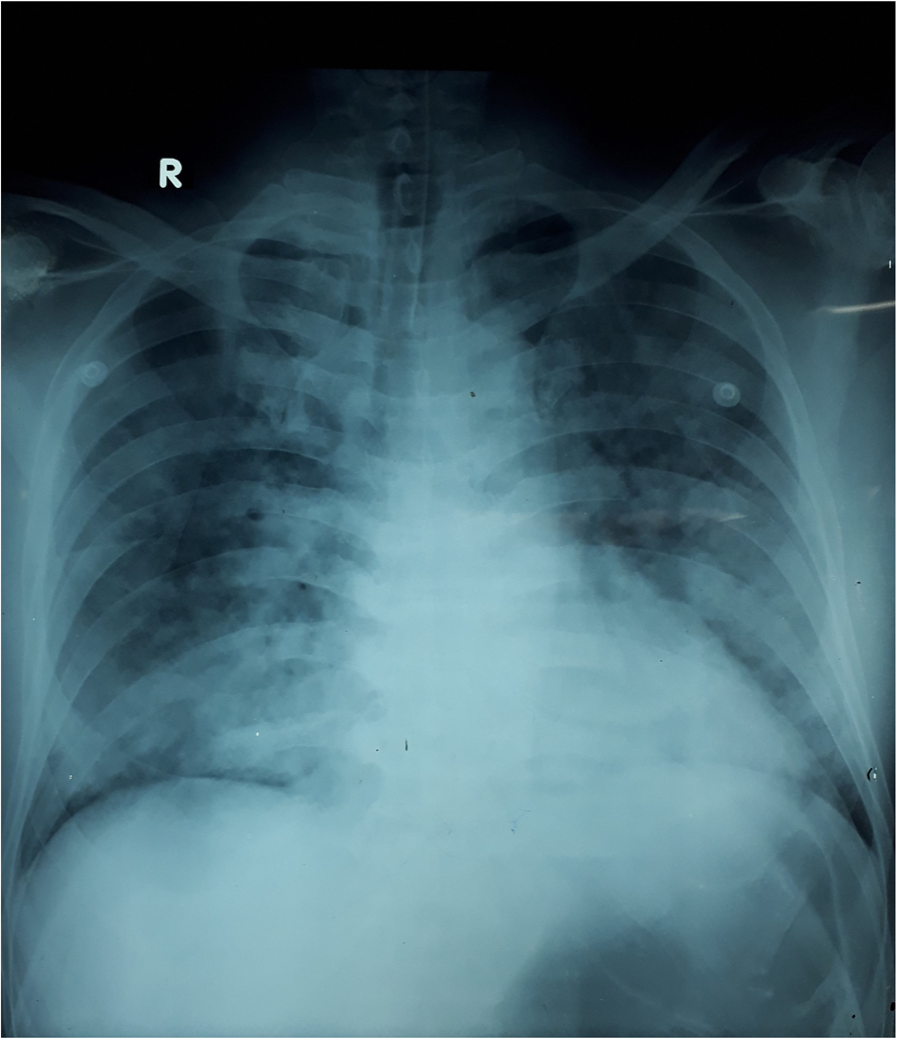
Bilateral alveolar shadowing suggestive of pulmonary haemorrhages

**Fig. 2:**
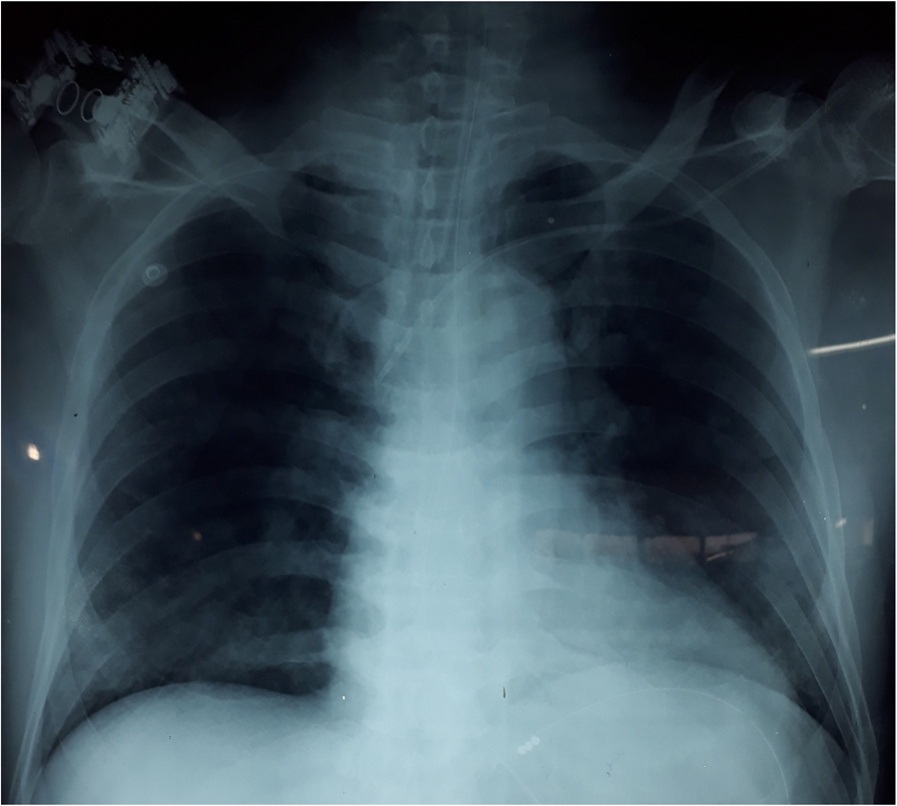
Resolution of pulmonary haemorrhage after steroid therapy

On the 3rd day, he became tachypneic with de-saturation and blood gases revealed PH 7.21, PCO_2_ 45 mmHg, PO2 31 mmHg HCO_3_ 12.4 mEq/L. He was intubated and started on mechanical ventilation. Bleeding through the endotracheal tube was noted but there was no bleeding from elsewhere. Chest x-ray revealed bilateral alveolar shadowing suggestive of pulmonary haemorrhages (Fig. 1). At this time his platelet count was 56 × 10^9^/L, INR 1.1, APTT 40 s, thrombo-elastometry showed only a deficiency of platelets. Due to the life-threatening nature of the situation, he was commenced on intravenous methylprednisolone 1 g pulse therapy daily along with FFP and platelet transfusions. There was a rapid improvement of hypoxia with the resolution of chest x-ray changes during the next 48 h. We discontinued steroid therapy after 3 days as there was no further bleeding and chest x-ray changes were resolving (Fig. 2).

**Fig. 3:**
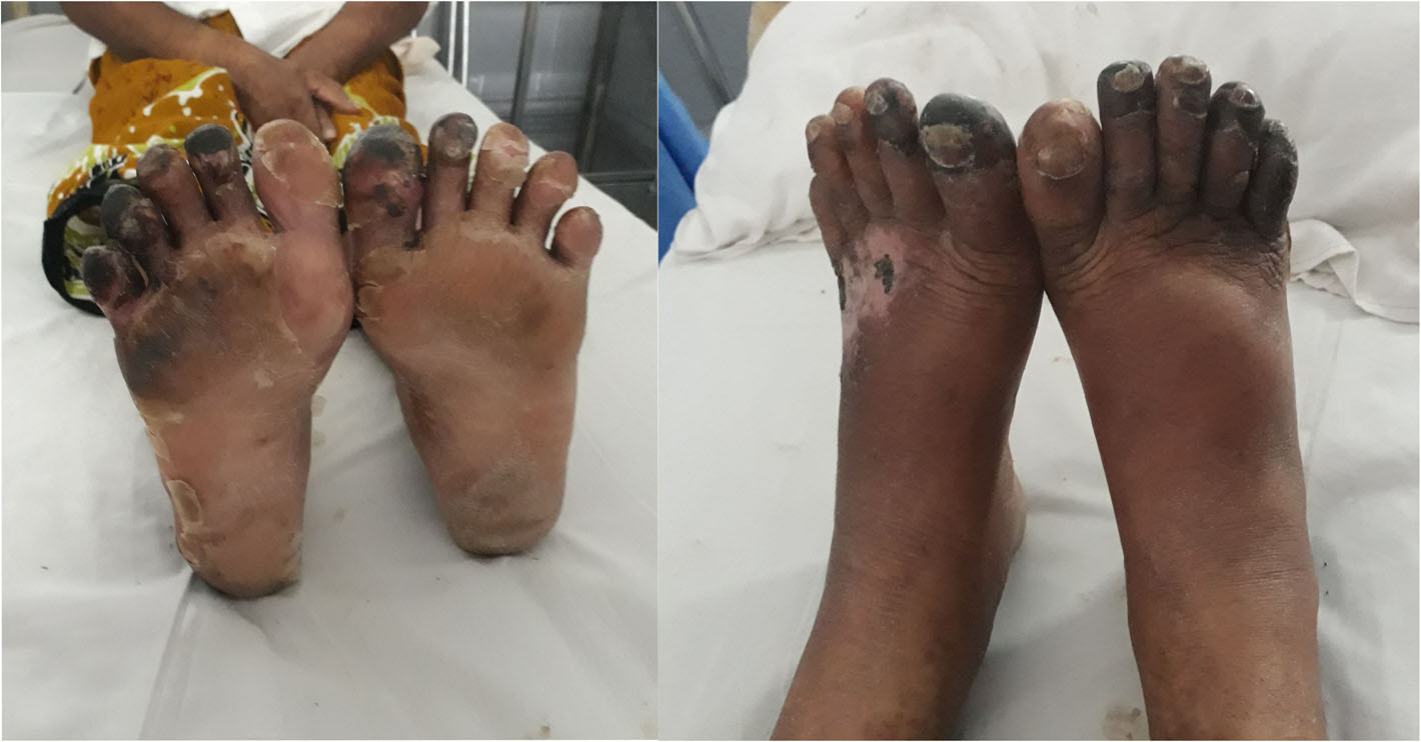
Dry gangrene of toes on both feet

Because of, persistent TMA as evidenced by a further drop in haemoglobin (8 mg/dl) and platelets (28 × 10^9^/L) plasmapheresis was commenced and continued for 6 cycles. Despite the effective treatment of TMA, the patient went on to develop dry gangrene of toes on both feet (Fig. 3). Renal function did not improve and required long term maintenance haemodialysis. He underwent renal transplantation 11 months after the incident due to end-stage renal disease.

## Discussion and conclusions

The commonest cause of snake bite envenoming in Sri Lanka is due to the hump-nosed viper [1, 2]. Three species of HNV of the genus *Hypnale* are found in Sri Lanka; *Hypnale hypnale, H. nepa and H. zara* [4]. Previously regarded as a mildly poisonous snake, hump-nosed viper is now known to cause significant systemic toxicity and fatalities. Maduwage et al. demonstrated potent cytotoxic, weak procoagulant, neurotoxic, myotoxic and phospholipase A2 activities in all three *Hypnale* venoms in vitro [5]. Clinical manifestations of envenomation are the pain, local swelling, occasionally haemorrhagic blisters at the bitten site, coagulopathy including TMA, and AKI. Coagulopathy is reported in 3.8% of cases which occurs within the first 24 h [6].

Identification of the snake by the victim or by medical personnel is the commonest means of diagnosing the respective snake. When the snake is unidentified visually, a syndromic diagnosis can be made in the clinical setting by applying clinical features into a validated algorithm [7]. Mild local swelling, blister formation and renal failure were the key clinical features in this patient favouring HNV bite. Twenty-minute blood clotting time can be normal in the first 24 h. The absence of neurological features helped to differentiate from toxicity due to Russell viper bite. Later development of coagulopathy and TMA further confirmed the HNV toxicity.

The fact that this patient’s pulmonary haemorrhage occurred with mild coagulopathy suggests that it is due to a direct effect of venom rather than due to coagulopathy. Studies in mice have shown that hump-nosed venom can induce macroscopic pulmonary congestion, oedema, gross haemorrhages and petechial haemorrhages [2]. *H.hypnale* causes petechial haemorrhages which were observed microscopically with a minimum amount of venom [8]. It is known that metalloproteinases found in the snake venom can induce the release of inflammatory mediators such as cytokines which intensify the inflammatory response. Metalloproteinases comprise a series of zinc-dependent enzymes, of varying molecular mass, which plays a key role in the haemorrhagic effects by acting directly on the capillary basement membrane and endothelial cells [9].

Although a single case of death due to acute kidney injury and coagulopathy leading to death following *H. zara* envenoming is reported in the literature, we did not find any reported cases of pulmonary haemorrhage due to hump-nosed viper bite. Almost all cases of pulmonary haemorrhage have been reported for *Bothrops* species bites which are endemic to Central and South America. *Bothrops* venom has both procoagulant and anticoagulant properties and pulmonary haemorrhage is secondary to anticoagulant properties [10]. There was only one case of a fatal outcome due to pulmonary haemorrhage following Russel’s viper bite reported from Sri Lanka [11]. This patient continued to bleed into the lungs despite normalization of clotting parameters and anti-venom therapy. In a large case series of Russell’s viper bites described (336 patients) by Kularatne et al. [12], although 77% of patients had evidence of coagulopathy as demonstrated by a nonclotting 20-min whole-blood clotting test, none showed frank pulmonary haemorrhage. Disseminated intravascular coagulopathy was observed only in seven patients (2%).

Pulmonary haemorrhage is a rare complication of snake bite [13]. This is the first case reported from humped-nose viper bite, which was diagnosed based on the clinical criterion. Furthermore, other common instances where IV methylprednisolone is given are pulmonary haemorrhages due to vasculitis, connective tissue disorders and leptospirosis etc. Our experience in using methylprednisolone in severe leptospirosis with pulmonary haemorrhages encouraged us to use it in this patient with an excellent response.

Life-threatening complications of HNV bite is usually managed with supportive treatment as specific anti-venom is not available for hump-nosed viper envenomation. Thus, our case report can contribute to open further clinical trials to develop antivenom and to implement our management with steroids in pulmonary haemorrhage.

Pulmonary haemorrhages with HNV bite are extremely rare and occurs due to direct toxicity rather than coagulopathy. It is associated with increased mortality and morbidity. Early consideration of pulmonary haemorrhages in a hypoxic patient led to treatment with steroids with a successful outcome and early weaning off from the mechanical ventilation.

## Data Availability

All data supporting our findings is contained within the manuscript.

## Abbreviations

CSTH: Colombo South teaching hospital
HNV: Hump-nosed viper
MAHA: Micro angiopathic haemolytic anaemia
